# Protective Effects of Dexmedetomidine Against Ischemic Heart Disease and Diabetic Cardiomyopathy by Targeting Ferroptosis

**DOI:** 10.31083/RCM45991

**Published:** 2026-06-08

**Authors:** Li Ren, Dongqing Li, Yuan Gao

**Affiliations:** ^1^Department of Anesthesiology, Zhuhai People's Hospital, The Affiliated Hospital of Beijing Institute of Technology, Zhuhai Clinical Medical College of Jinan University, 519000 Zhuhai, Guangdong, China

**Keywords:** cardiovascular diseases, ferroptosis, dexmedetomidine, ischemic heart disease

## Abstract

Cardiovascular diseases (CVDs) are the leading cause of morbidity and mortality worldwide. Ischemic heart disease and diabetic cardiomyopathy are two CVDs characterized by prominent myocardial injuries. Calcium overload, mitochondrial damage, the accumulation of reactive oxygen species (ROS), and abnormal programmed cell death (PCD) pathways, such as autophagy, pyroptosis, apoptosis, and ferroptosis, are recognized as the major mechanisms of myocardial injury. Dexmedetomidine (DEX) is a selective α2-adrenergic receptor agonist that is often administered to surgical patients due to the associated sedative, analgesic, and anxiolytic properties. Recent studies have indicated that DEX can exhibit more beneficial effects in patients, including reducing myocardial and vascular damage in CVD patients. Mechanistically, DEX reduces levels of oxidative stress markers and inflammatory cytokines and improves mitochondrial function. Numerous studies have revealed novel regulatory roles for DEX in mediating ferroptosis. This study summarizes the expression and functions of ferroptosis in ischemic heart disease and diabetic cardiomyopathy and discusses the regulatory mechanism of DEX in ferroptosis.

## 1. Introduction

Cardiovascular diseases (CVDs) remain a leading cause of mortality worldwide, a trend exacerbated by population aging and the COVID-19 pandemic [1]. Between 2025 and 2050, the incidence of CVD is projected to increase by 90.0%, with crude mortality increasing by 73.4% and crude disability-adjusted life years increasing by 54.7% [2]. Ischemic heart disease (IHD), the most prevalent CVD, has experienced dramatic growth in the number of deaths worldwide. IHD-related deaths increased from 2 million in 2000 to 8.9 million in 2019, accounting for 16% of total global deaths that year [3]. Diabetic cardiomyopathy (DCM), one of the leading causes of death in people with diabetes, also has an increased incidence as diabetes progresses [4]. The complex pathophysiology of both IHD and DCM involves the accumulation of calcium ions and reactive oxygen species (ROS), mitochondrial dysfunction, endothelial damage, and immune activation, which trigger various forms of programmed cell death (PCD) pathways, such as autophagy, apoptosis, necroptosis, and pyroptosis [5, 6]. Ferroptosis, an iron-dependent lipid peroxidation-driven mechanism, fundamentally results from oxidative imbalance and iron homeostasis disruption, leading to membrane structural damage [7]. Ferroptosis, an essential type of PCD, plays a significant role in the pathophysiology of both IHD and DCM, and targeting ferroptosis can have significant protective effects [8].

Dexmedetomidine (DEX) is a selective α-2-adrenergic receptor agonist that is often administered to surgical patients due to the associated sedative, analgesic, and anxiolytic properties. DEX has a protective effect on a variety of organs, including the nervous system, heart, lungs, kidneys, liver, and small intestine [9]. Current studies have shown that DEX has protective effects on CVDs, including cardiac arrest [10], myocardial infarction [11], and DCM [12]. In terms of mechanism, DEX has prominent anti-inflammatory, antiapoptotic, antioxidative, and antifibrotic effects [13]. Recently, DEX was shown to promote nuclear factor erythroid 2-related factor 2 (Nrf2), solute carrier family 7 member 11 (SLC7A11), ferroptosis suppressor protein 1 (FSP1), coenzyme Q10 (CoQ10), and glutathione peroxidase 4 (GPX4), which are vital mediators of the ferroptosis process [14]. An increasing number of studies have revealed the essential roles of DEX-mediated protection against IHD through the targeting of ferroptosis-related mediators. This article summarizes the alterations in ferroptosis in IHD and DCM and the functions and mechanisms by which DEX regulates ferroptosis to exert protective effects on IHD and DCM, aiming to provide new therapeutic targets and strategies for the prevention and treatment of CVD.

## 2. Mechanisms of Ferroptosis in IHD

### 2.1 Overview of the Main Mechanisms of Ferroptosis

**Fig. 1.**
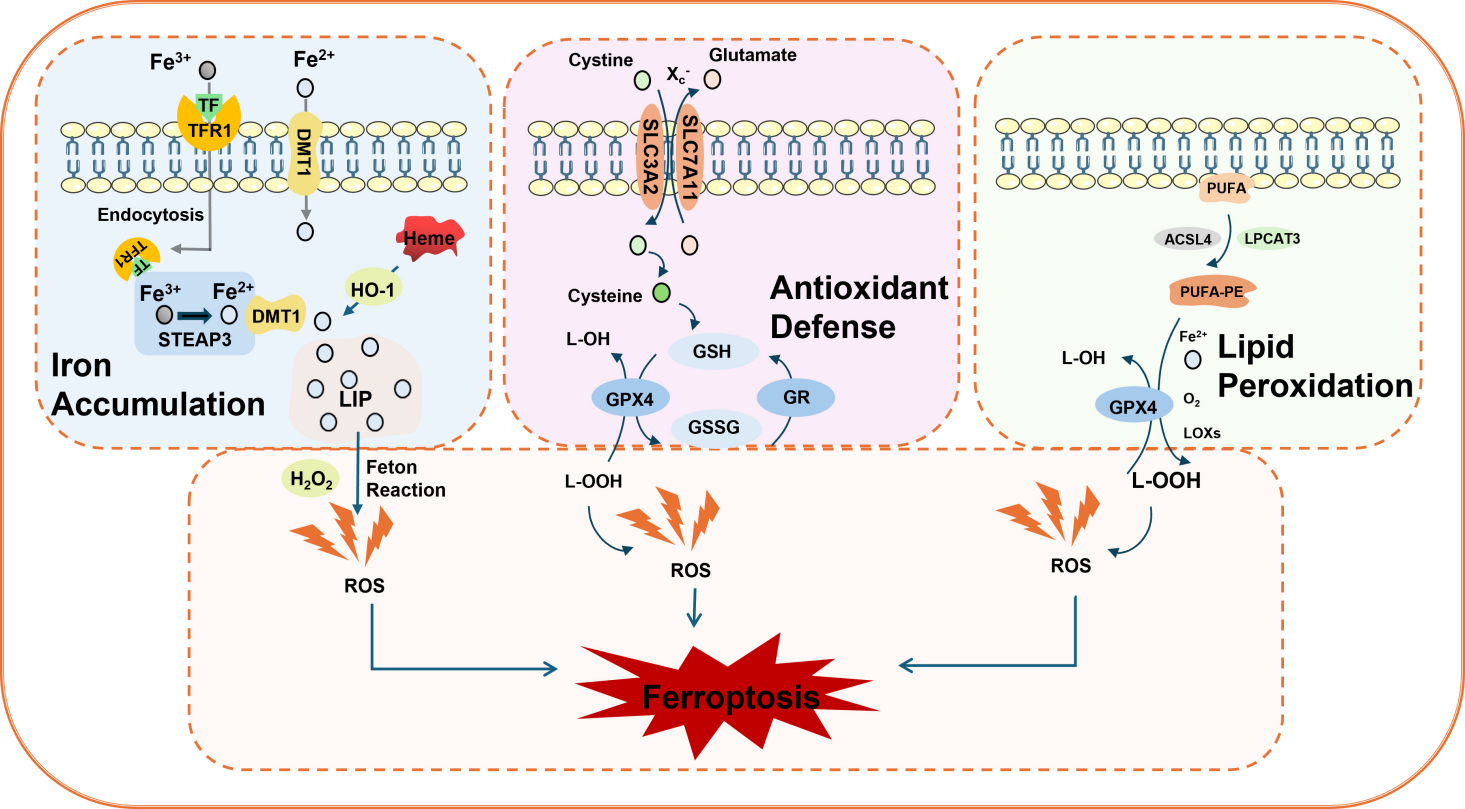
**Schematic diagram of ferroptosis**. Iron accumulation: Transferrin receptor 1 (TFR1) transports extracellular ferric ions (Fe^3+^) into cells through endocytosis. These ions are then converted to ferrous ions (Fe^2+^) via the mediation of the metalloproteinase six-transmembrane epithelial antigen of prostate 3 (STEAP3) and divalent metal transporter 1 (DMT1), which are subsequently released into the cytoplasm. Heme oxygenase 1 (HO-1) releases Fe^2+^ from heme, further exacerbating intracellular iron accumulation. Antioxidant system: The cystine–glutamate cotransporter (X), which is composed of solute carrier family 7 member 11 (SLC7A11) and SLC3A2C^–^, an extracellular cysteine, enters the cytoplasm while glutamate is transported to the extracellular space. Intracellular cysteine is reduced to cysteine, which serves as a rate-limiting precursor for glutathione (GSH) synthesis. As a reducing substrate, GSH is catalyzed by glutathione peroxidase 4 (GPX4) to convert lipid peroxides (L-OOH) into nontoxic lipoyl alcohol (L-OH) while being oxidized into oxidized glutathione (GSSG). This process is crucial for scavenging excess peroxides and hydroxyl radicals. When GSH depletion or GPX4 inhibition occurs, cells lose their ability to eliminate lipid peroxides. The Fe^2+^ generated through the Fenton reaction further exacerbates lipid peroxidation, accelerating the ferroptosis process. Lipid peroxidation: Long-chain acyl-CoA synthase 4 (ACSL4) catalyzes the binding of polyunsaturated fatty acids (PUFAs) with phosphatidylethanolamine (PE) via lysophosphatidyltransferase 3 (LPCAT3), forming PUFA-PE. Lipoxygenase (LOX) oxidizes both free and esterified PUFAs, producing lipid signaling molecules and reactive oxygen species (ROS) that directly drive the accumulation of lipid peroxides.

Ferroptosis is characterized by lipid peroxidation and an excess iron load [7]. Its morphological features primarily include mitochondrial shrinkage, increased mitochondrial membrane density, disruption of mitochondrial cristae, and outer membrane rupture, whereas changes in nuclear morphology are less prominent. The classic mechanisms of ferroptosis involve iron ion metabolic imbalance, redox disorders, and lipid peroxidation [15] (Fig. 1).

During iron metabolism, transferrin receptor 1 (TFR1) transports extracellular ferric ions into cells through endocytosis. Under the mediation of six-transmembrane epithelial antigen of prostate 3 (STEAP3) and divalent metal transporter 1 (DMT1) (disulfide-binding reductase), ferric ions are converted to ferrous ions, which are released into the cytoplasm to initiate the Fenton reaction [16]. Simultaneously, ferrous ions can be released from heme via heme oxygenase 1 (HO-1), thereby accelerating ferroptosis [17].

The core antioxidant system involves the cystine‒glutamate antiporter (XC)-glutathione (GSH)-GPX4 axis. First, XC^–^ transporter proteins (composed of SLC7A11 and SLC3A2 subunits) facilitate the uptake of extracellular cysteine into the cytoplasm for glutamate synthesis, subsequently transporting glutamate to the extracellular space [13, 18]. During this process, intracellular cysteine is reduced to cysteine, which serves as a rate-limiting precursor for GSH synthesis. GSH subsequently acts as a reducing substrate. Under the catalysis of GPX4, lipid peroxides (L-OOH) are reduced to nontoxic lipid alcohol (L-OH), while GSH itself is oxidized to glutathione disulfide (GSSG). This mechanism effectively eliminates excess peroxyl radicals and hydroxyl radicals generated during cellular respiration and metabolic processes. GPX4 activity is crucial for inhibiting lipid peroxidation. When GSH depletion or GPX4 inhibition occurs, divalent iron triggers lipid peroxidation through the Fenton reaction, generating substantial amounts of ROS that promote ferroptosis [19].

Polyunsaturated fatty acids (PUFAs), the primary components of cell membrane phospholipids, exhibit metabolic abnormalities closely associated with ferroptosis [20]. Lipid peroxidation serves as the central mechanism in ferroptosis, where PUFAs are oxidized into lipid peroxides through specific enzymatic or radical reactions. Long-chain acyl-CoA synthase 4 (ACSL4) catalyzes the binding of PUFAs with CoA to form PUFA-CoA. This compound then interacts with phosphatidylethanolamine (PE) via lysophosphatidyltransferase 3 (LPCAT3), forming the polyunsaturated fatty acid phosphatidylethanolamine (PUFA-PE) [13, 21]. Lipoxygenase (LOX), a class of iron-containing enzymes, catalyzes both the oxidation of free and esterified polyunsaturated fatty acids (FAs), generating lipid signaling molecules and ROS [22]. Additionally, ROS can react with polyunsaturated fatty acids in lipid membranes, triggering lipid peroxidation. When environmental Fe^2+^ levels are excessive, this reaction is significantly enhanced through the Fenton mechanism. Metabolic byproducts of lipid peroxidation, such as malondialdehyde (MDA) and 4-hydroxynonenic acid (4-HNE), disrupt biological membrane integrity by altering their physical properties—such as reducing permeability and fluidity—ultimately leading to cell death [23].

### 2.2 Changes in Ferroptosis in IHD and DCM

The cardiovascular system requires iron to meet its high energy demands and metabolic activities. Iron plays a vital role in oxygen transport and storage, mitochondrial function, and enzyme activity. However, excessive iron also causes cardiotoxicity, as it can contribute to excessive ROS and oxidative damage [24]. Alterations in iron metabolism and ferroptosis-related biomarkers in CVD, such as IHD and DCM, are gaining increasing attention.

Following acute myocardial infarction (AMI), MDA levels are significantly increased, whereas the levels of antioxidants such as GSH, superoxide dismutase (SOD), and GPx are reduced [25]. Multiple studies have shown that patients with diabetic myocardial infarction (DMI) and those with isolated myocardial infarction exhibit significantly elevated levels of MDA, while the levels of the antioxidant proteins SOD and GSH are markedly reduced [26], indicating that the diabetic background further promotes oxidative reactions. Ho *et al*. [27] reported that the levels of serum ACSL4 and the proinflammatory cytokine IL-1β were significantly greater in patients with ST-segment elevation myocardial infarction (STEMI) than in healthy controls. Multivariate logistic regression analysis revealed that ACSL4 levels were an independent risk factor for cardiovascular adverse events within one year of STEMI, suggesting its potential as a biomarker for poor prognosis in STEMI patients. Jia *et al*. [28] determined the serum levels of GPX4, ferritin heavy chain 1 (FTH1), FSP1, and 4-HNE in patients with STEMI before primary percutaneous coronary intervention (pPCI) and at 6 h after pPCI. They reported that ferroptosis-associated mediators were not significantly altered at 6 h following pPCI, whereas nicorandil administration increased GPX-4, FTH1, and FSP1 levels and inhibited 4-HNE release [28].

In IHD and DCM, the role of ferroptosis in cardiomyocytes has been well studied and reviewed in previous studies [5, 6, 7, 8]. Notably, multiple cellular components, including endothelial cells (ECs), monocytes/macrophages, and vascular smooth muscle cells (VSMCs), exhibit dynamic changes in ferroptosis. Single-cell RNA sequencing (scRNA-seq) analysis of AMI revealed that the number of ECs, the largest population of nonmyocytes within myocardial tissue, was reduced. These genes significantly increased the expression of ferroptosis-related genes [29]. Yin *et al*. [30] reported that guanylate cyclase soluble subunit alpha 1 (GUCY1A1) was downregulated in ECs. Specific knockout of GUCY1A1 in ECs contributed to increased ferroptosis and enhanced microvascular disorders on day 3 following I/R injury. When GUCY1A1 was specifically knocked out in VSMCs, reduced arteriogenesis and poor cardiac functions were found on day 28 post-I/R injury [30]. Atherosclerosis is a significant pathological basis of CVD, especially IHD. Compared with those in stable plaques, human atherosclerotic artery tissues with unstable plaques presented reduced VSMC and GPX4 expression and increased infiltration of CD68^+^ macrophages. The expression of the gene encoding YAP1, which is enriched in VSMCs within atherosclerotic plaques, was reduced in VSMCs from high-fat diet (HFD)-fed *Apoe*^-⁣/-^ mice. Suppression of ferroptosis improved atherosclerotic plaque stability, and overexpression of YAP1 mitigated ferroptosis in VSMCs, suggesting that targeting YAP1 significantly reversed VSMC dysfunction by preventing ferroptosis [31]. Within cardiac tissues, macrophages are composed of resident macrophages [without C-C chemokine receptor 2 (CCR2) expression] and recruited circulating monocytes [CCR2+ positive cells, also termed monocyte-derived macrophages (MoMs)]. AMI causes the death of resident macrophages, while MoMs are recruited into the ischemic myocardium and function as the main phagocytes [32]. In the AMI model, MoMs enhanced ferroptosis and reduced efferocytosis. Piezo1, a mechanosensitive channel that significantly affects ferroptosis, has elevated expression in MoMs stimulated by oxygen‒glucose deprivation (OGD). Piezo1 deficiency or SLC7A11 knockdown suppressed ferroptosis and promoted efferocytosis of MoMs, thus mitigating cardiomyocyte damage and improving left ventricular remodeling and cardiac functions in an AMI model [33]. Overall, targeting ferroptosis can alleviate the dysfunction of multiple cellular components in CVD.

## 3. Effects of DEX on IHD and DCM via the Inhibition of Ferroptosis

**Fig. 2.**
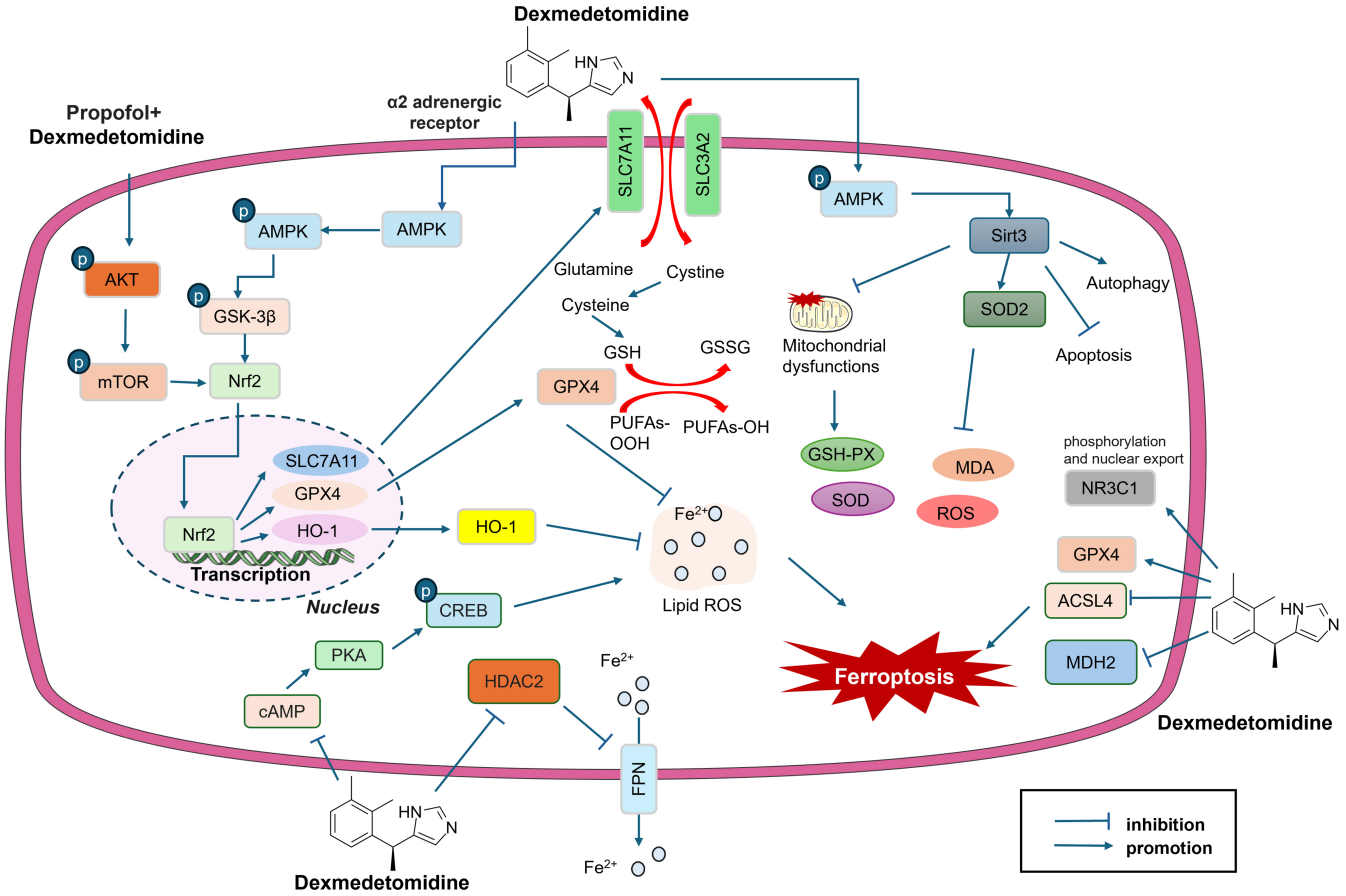
**DEX regulates ferroptosis in IHD and DCM**. DEX affects multiple signaling pathways, including the Nrf2, AMPK, GSK-3β, Akt-mTOR, cAMP/PKA/CREB, and Sirt3 pathways. DEX enhances SLC7A11, GPX4, and FPN but represses ACSL4. Therefore, DEX attenuates MDA, ROS, and Fe^2+^ levels, improves mitochondrial dysfunction, and promotes GSH and SOD generation. DEX, dexmedetomidine; IHD, ischemic heart disease; DCM, diabetic cardiomyopathy; Nrf2, nuclear factor erythroid 2-related factor 2; AMPK, AMP-activated protein kinase; cAMP, cyclic adenosine monophosphate; PKA, protein kinase A; CREB, cAMP response element-binding protein; SLC7A11, solute carrier family 7 member 11; GPX4, glutathione peroxidase 4; FPN, ferroportin; ACSL4, long-chain acyl-CoA synthase 4; MDA, malondialdehyde; ROS, reactive oxygen species; GSH, glutathione; SOD, superoxide dismutase.

The pathogenesis of IHD and DCM involves multiple mechanisms, such as coronary artery disease, thrombosis, vascular blockage, and myocardial damage. Restoring coronary blood flow promptly constitutes the core therapeutic strategy for AMI. Research has confirmed that DEX can modulate key pathophysiological processes in IHD and DCM by mediating ferroptosis (Table 1, Ref. [34, 35, 36, 37, 38, 39, 40, 41, 42, 43, 44, 45, 46, 47, 48], Fig. 2).

**Table 1.** **Mechanisms by which dexmedetomidine targets ferroptosis in CVD patients**.

Heart diseases	Model	Functions and mechanisms	Changes of ferroptosis-associated mediators	Ref
IHD	MIRI	alleviate myocardial infarction, improve heart function, promote the AMPK/GSK-3β/Nrf2 axis	Fe^2+^↓ lipid peroxidation↓ SLC7A11↑ GPX4↑↓	[34]
	H/R	inhibit myocardial IRI by activating the Nrf2/Sirt3/SOD2 signaling pathway to suppress oxidative stress	ROS↓ MDA↓ SOD↑ GSH↑	[35]
	H/R	improve cardiac dysfunction and alleviate oxidative damage caused by H/R by upregulating the transcription of FPN	FPN↑	[36]
	I/R	reduce lipid accumulation and infarct volume caused by myocardial I/R damage, and inhibit the NF-κB pathway	MDA↓ SOD↑ GSH↑	[37]
	I/R	improve morphology and myocardial ultrastructure, attenuate oxidative stress and inflammation, activate the Nrf2 pathway	SLC7A11↑ GPX4↑ Ferritin↓ TFR1↓ ACSL4↓	[38]
	I/R	reduce production of cell-damaging free radicals; alleviate myocardial IRI	ROS↓ SLC7A11↑ GPX4↑ FTH↑	[39]
	MIRI	alleviate myocardial injury; upregulate NR3C1 phosphorylation; downregulate PDK4; reduce lactate production; improve mitochondrial function	GSH↑ MDA↓	[40]
	H/R	DEX and propofol combination alleviates the cardiac dysfunction and myocardial cell damage caused by MIRI; activates the Akt/mTOR and Nrf2/GPX4 signaling pathways	GPX4↑ SOD↑ MDA↓	[41]
	H/R	upregulate the expression of miR-141-3p; downregulate the expression of lncRNA TUG1; inhibit oxidative stress	GSH↑ GPX4↑ ACSL4↓	[42]
	I/R	improve mitochondrial ultrastructure and restore ATP production in I/R hearts; promote autophagy; activate AMPK/Sirt3 pathway; inhibit the release of pro-inflammatory factors	ROS↓ MDA↓ SOD↑ GSH↑	[43]
	I/R	enhance the expression of PKA and the phosphorylation of CREB and ERK1/2; inhibit oxidative stress; prevent myocardial injury	SOD↑ GPX4↑	[44]
DCM	H9C2 cells induced with high glucose	upregulate GPX4; promote the nuclear translocation of Nrf2; activate the Nrf2/GPX4 pathway; reduce oxidative stress	GPX4↑ MDA↓ ROS↓ Fe^2+^↓	[45]
DIC	Adriamycin-induced cardiotoxicity model	improve cardiac functions; reverse the upregulation of ANP, BNP, MHC, and Collagen III protein levels; increase the expression of Nrf2 in the nucleus; activate the Akt/GSK-3β signaling axis	ROS↓ MDA↓ FTH1↑ GSH↑ GPX4↑ SLC7A11↑	[46]
Sepsis-induced myocardial injury	CLP	inhibit myocardial injury and inflammatory factors (IL-6 and monocyte chemoattractant protein-1)	MDA↓ GPX4↑ GSH↑	[47]
	LPS treatment	alleviate PRMT5 expression, inflammation, and myocardial injury in septic mice	ROS↓ MDA↓ SOD↑ GSH↑ GPX4↑ SLC7A11↑	[48]

Notes: ACSL4, long-chain acyl-CoA synthase 4; AMPK, AMP-activated protein kinase; CREB, cAMP response element-binding protein; CLP, cecal ligation and puncture; DIC, drug-induced cardiotoxicity; DCM, diabetic cardiomyopathy; ERK 1/2, extracellular signal regulated kinase 1/2; FPN, ferroportin; FTH, ferritin heavy chain; GPX4, glutathione peroxidase 4; H/R, hypoxia/reoxygenation; IHD, ischemic heart disease; LPS, lipopolysaccharide; MDA, malondialdehyde; MIRI, myocardial infarction-reperfusion; Nrf2, nuclear factor erythroid 2-related factor 2; PDK4, pyruvate dehydrogenase kinase 4; PKA, protein kinase A; PRMT5, protein arginine methyltransferase 5; ROS, reactive oxygen species; Sirt, sirtuin; TUG1, taurine upregulated 1; GSK-3β, Glycogen synthase kinase 3β.

### 3.1 DEX Mediates Protective Effects in IHD by Inhibiting Ferroptosis

In myocardial infarction-reperfusion injury (MIRI) models, DEX treatment significantly dampened myocardial infarction size and improved heart function [34]. Notably, DEX shows protective effects against MIRI, whether by preconditioning or postconditioning, in MIRI animal models [35, 38]. In hyperlipidemic rats subjected to MIRI, DEX administration improved cardiac function and mitigated the expression of cardiac injury indicators and oxidative stress indicators (MDA, SOD, and GSH) [37].

DEX has regulatory effects on multiple mediators that are involved in ferroptosis: (1) Fe^2+^; (2) core proteins of the ferroptosis process, which include ferritin, GPX4, ACSL4, SLC7A11, FTH, TFR1, and ferroportin (FPN); and (3) ROS and oxidative stress indicators (including MDA, SOD, and GSH-PX) (Table 1). For example, DEX preconditioning in male Sprague‒Dawley rats reduced infarct size; increased the expression of GPX4 and SLC7A11; repressed the expression of ferritin, TFR1, and ACSL4; and reduced the production of inflammatory cytokines (IL-1β, IL-6, and TNF-α). The administration of yohimbine, an α2 adrenergic receptor antagonist, can reverse the protective effects of DEX [38]. As an iron uptake protein, TFR1 transports transferrin-bound iron into cells. Upregulated TFR1 enhances iron uptake, leading to iron overload, indicating that ferroptosis is activated after MIRI. DEX treatment mitigates these adverse effects by suppressing TFR1 upregulation and reducing the intracellular iron content [38].

In myocardial injury, excessive iron accumulation promotes lipid peroxidation. ACSL4, a key regulator of the ferroptosis pathway, participates in the synthesis of polyunsaturated fatty acids associated with lipid peroxidation. In the I/R mouse model, elevated ACSL4 protein expression was detected, but this change was reversed by DEX [42].

FPN1 is considered a vital nonheme cellular iron export protein that is responsible for the export of iron from iron storage cells into the blood. Reduced FPN1 expression or FPN1 deletion can contribute to the accumulation of ROS and increased ferroptosis. Nrf2 can control the transcription of FPN1. Activation of the Nrf2/FPN1 axis markedly prevents MIRI in diabetic rats by suppressing Fe^2+^, SOD, MDA, and ACSL4 and enhancing GPX4 [49]. Fu *et al*. [36] established an *ex vitro* hypoxia/reoxygenation (H/R) model in cardiomyocytes and an MIRI model in male C57BL/6 J mice. They reported that DEX treatment inhibited H/R-mediated ferroptosis in cardiomyocytes and promoted FPN expression. Following FPN knockdown, the DEX-mediated protective effects were abolished. Histone deacetylase 2 (HDAC2) is a key subtype of histone deacetylase (HDAC) enzymes responsible for deacetylating histones H3 and H4. HDAC2 was promoted in H/R-induced cardiomyocytes. DEX can inhibit HDAC2 expression, and the knockdown of HDAC2 relieved MIRI and ferroptosis in mice. Moreover, H/R-induced HDAC2 upregulation disturbed FPN expression at the transcriptional level by inhibiting the Histone H3 lysine 27 acetylation (H3K27Ac) level in the FPN promoter region. Rescue experiments revealed that HDAC2 overexpression partially dampened the effects of DEX on H/R-mediated cardiomyocyte ferroptosis [36].

Previous studies have verified that ferroptosis is closely related to mitochondrial function. The stimulation of hypoxia and ischemia contributes to significant mitochondrial dysfunctions (including imbalances in mitochondrial fusion and fission, dysregulated mitophagy, decreased mitochondrial membrane potential, and mitochondrial Ca^2+^ overload) in the heart, which then promote oxidative stress and induce ferroptosis [50]. Interestingly, studies have shown that DEX can reverse mitochondrial dysfunction in IHD. Yu *et al*. [39] reported that DEX treatment reduced IRI-induced myocardial injury, alleviated mitochondrial dysfunction, decreased the level of ROS, alleviated mitochondrial dysfunction, inhibited the activation of SLC7A11/GPX4, and modulated the expression of ferroptosis-related proteins, including SLC7A11, glutathione peroxidase 4 (GPX4), FTH, and cyclooxygenase-2 (COX-2). Conversely, the ferroptosis activator erastin partly suppressed DEX-mediated cardioprotection. Taken together, these results reveal that DEX inhibits ferroptosis by increasing the expression of SLC7A11 and GPX4, thereby preventing cardiac I/R injury.

### 3.2 Signaling Pathways Mediated by DEX in the Modulation of Ferroptosis

Multiple signaling pathways are involved in the DEX-mediated cardioprotective effects on the regulation of ferroptosis (Fig. 2).

As a vital transcription factor, Nrf2 protects cells from oxidative stress and maintains cellular redox homeostasis. It also plays a role in regulating various ferroptosis-related proteins (GPX4, SLC7A11, HO-1, etc.). DEX activates Nrf2, thereby promoting SLC7A11 and GPX4 expression to inhibit ferroptosis and effectively reduce MIRI in mice [34]. H9C2 cells treated with high glucose (HG) presented significant decreases in cell viability and apoptosis and increased levels of Fe^2+^, MDA, and ROS. The protein levels of nuclear Nrf2 and GPX4 were also repressed. DEX administration reversed HG-induced H9C2 cell damage by reducing apoptosis and increasing the nuclear translocation of Nrf2, thus promoting GPX4 expression. However, DEX-mediated protective effects are partially disrupted by the inhibition of Nrf2 [45]. The protective effects of the DEX-mediated Nrf2 pathway in IHD have been verified in various studies (Table 1). In the DCM rat model, the expression of Nrf2 in myocardial tissues was significantly reduced. Cardiomyocyte-specific overexpression of Nrf2 attenuated Fe^2+^ levels and Fe^3+^ deposition and restrained MDA-mediated lipid peroxidation. In addition, Nrf2 overexpression promoted the protein levels of SLC7A11, GPX4, and FTH1, suggesting that Nrf2 can directly prevent ferroptosis in DCM [51].

AMP-activated protein kinase (AMPK) is the primary sensor of cellular energy through adenine nucleotide levels and plays a major role in regulating the cellular energy balance. Many studies have revealed that DEX can activate the AMPK pathway, which is often repressed following IHD [34, 43]. For example, the phosphorylation levels of both AMPK and Glycogen synthase kinase 3β(GSK-3β) were reduced in H/R-treated H9c2 cells. DEX treatment increased p-AMPK and p-GSK-3β levels and promoted the nuclear translocation of Nrf2. The administration of an AMPK agonist (AICAR) enhanced DEX-mediated Nrf2 upregulation, whereas an AMPK inhibitor (Compound C, CC) reversed DEX-mediated nuclear translocation of Nrf2 [34]. In addition, Sirt3, a vital member of the sirtuin family, is also regulated by AMPK signaling and has significant effects on CVDs by mediating ferroptosis [52]. For example, p-AMPK, PGC-1α, and Sirt3 levels were reduced in the hearts of the MIRI model, whereas MDA and GSSG levels were significantly increased, whereas CAT and GSH levels were markedly reduced. The administration of gypensapogenin I (GI) markedly increased p-AMPK, PGC-1α, and Sirt3 levels and suppressed ferroptosis by directly targeting NADPH oxidase 2 (NOX2). The protective effects of GI against ferroptosis and apoptosis were reversed following treatment with the AMPK inhibitor Compound C. Compound C also reduced PGC-1α and Sirt3 expression [52]. Hu *et al*. [35] reported that DEX can prevent MIRI by upregulating Nrf2 and Sirt3, thus relieving ferroptosis in MIRI hearts. He *et al*. [43] reported that DEX improved mitochondrial function and mitigated ferroptosis in oxygen‒glucose deprivation/reoxygenation (OGD/R)-treated H9c2 cells and that DEX promoted the recovery of myocardial damage and mitochondrial dysfunction in a MIRI mouse model. Mechanistically, DEX enhanced AMPK and Sirt3 expression in MIRI hearts, which was repressed following the administration of Compound C [43]. These studies suggest that DEX also plays a role in mediating ferroptosis via the AMPK-Sirt3 axis.

There are other signaling pathways involved in DEX-mediated cardioprotective effects, including the Akt/mTOR pathway [41] and the cyclic adenosine monophosphate (cAMP)/protein kinase A (PKA)/cAMP response element-binding protein (CREB) pathway [44]. For example, Yang *et al*. [41] constructed a rat model of MIRI and reported that the protein levels of p-Akt and p-mTOR were significantly decreased in the MIRI group. DEX treatment increased p-Akt and p-mTOR levels, accompanied by attenuated myocardial injury and MDA generation. mTOR directly binds Nrf2 and promotes its expression [41]. Ma *et al*. [44] reported that DEX prevents H/R-induced necrosis, apoptosis, and ferroptosis in H9c2 cells by restraining PKA, CREB, and extracellular signal-regulated kinase 1/2 (ERK1/2), indicating that the cAMP/PKA/CREB pathway plays a role in mediating ferroptosis [44]. cAMP/PKA signaling can modulate multiple cellular processes following AMI, including cardiac contractility, metabolism, gene expression, and mitochondrial dysfunction. Disruption of cAMP/PKA signaling by the overexpression of melanoma-associated antigen-A13 (Magea13) reversed MIRI- and OGD/R-induced H9c2 cell injury [53]. Previous studies have shown that DEX can directly suppress cAMP production via the α2-adrenergic receptor [54]. Overall, the DEX-mediated Akt/mTOR pathway and cAMP/PKA/CREB pathway are essential for mediating the ferroptosis process.

## 4. Conclusion and Future Perspectives

This study systematically elucidates the mechanisms of ferroptosis in CVD. DEX protects against IHD and DCM through mediating multiple signaling pathways (Fig. 2). Therefore, DEX increases the Nrf2/GPx4/GSH pathway and reduces Fe^2+^ accumulation and the levels of oxidative stress mediators in cardiomyocytes.

Although the protective effects of DEX have been confirmed in cardiomyocytes, its regulatory effects on other cellular compounds, such as ECs, monocytes/macrophages, and VSMCs, which undergo ferroptosis in IHD and DCM, remain to be explored in the future. In a FeCl3-induced carotid artery thrombosis mouse model, the mice presented increased thrombus weight and length and significant arterial endothelial loss. DEX pretreatment for 2 weeks reversed these alterations, which were dependent on α2-adrenergic receptor activation. This study suggested that DEX may mitigate stress-related thrombus formation in a mouse model and serve as a potential therapeutic strategy for chronic psychological stress-associated thrombotic events in patients with thrombotic CVDs [55].

Drug-induced cardiotoxicity (DIC) has been regarded as a major concern of drug-associated side effects. Ferroptosis plays an essential role in this pathological process [46]. For instance, propofol displays pronounced bidirectionality in the cardiovascular system. The combination of DEX with propofol achieved more significant protective effects against MIRI by activating the Akt/mTOR/Nrf2 signaling pathway and inhibiting ferroptosis-related molecules (e.g., downregulation of GPX4 and lipid peroxidation) [41]. In an Adriamycin-induced cardiotoxicity model, DEX administration improved cardiac function, reversed the upregulation of ferroptosis-associated mediators, increased the expression of Nrf2 in the nucleus, and promoted the activation of the Akt/GSK-3β signaling axis [46]. These studies indicate that combining DEX with other agents enhances its cardioprotective effects or reverses its cardiotoxic effects. However, the effects of DEX on the cardiovascular system require careful attention, particularly in children undergoing cardiac surgery. Its α2-adrenergic receptor agonist properties may significantly increase bradycardia risk by suppressing sympathetic activity and reducing catecholamine levels [56]. These adverse effects are more prevalent in elderly patients, those with concomitant conduction blocks, or those receiving rapid loading, necessitating rigorous clinical monitoring of vital signs to avoid combination therapy with opioid-like sympatholytics. The precise management of its side effects and the optimization of combination therapies still require further exploration to achieve optimal therapeutic outcomes and the best balance of safety.

## Abbreviations

4-HNE, 4-hydroxynonenic acid; α2-AR, α2-adrenergic receptor; ACSL4, long-chain acyl-CoA synthase 4; AIF, apoptosis-inducing factor; AIM2, absent in melanoma 2; AMI, acute myocardial infarction; AMPK, AMP-activated protein kinase; CABG, coronary artery bypass grafting; CAG, chronic atrophic gastritis; CFs, Cardiac fibroblasts; CHF, chronic heart failure; CRAD, caspase recruitment domain; CVDs, cardiovascular diseases; DAMPs, damage-associated molecular patterns; DCM, diabetic cardiomyopathy; DEX, dexmedetomidine; FBS, Fuster BEWAT score; Fer-1, ferrostatin-1; FPN, hepcidin-ferroportin; FTH-1, ferritin heavy chain 1; GPX4, Glutathione peroxidase 4; GSH, glutathione; GSSG, oxidized glutathione; HO-1, heme oxygenase 1; H/R, hypoxia/reoxygenation; ICU, intensive care unit; IHD, ischemic heart disease; I/R, ischemia/reperfusion; L-OH, lipid alcohol; L-OOH, lipid peroxides; LOX, Lipoxygenase; LPCAT3, lysophosphatidyltransferase 3; LPS, lipopolysaccharide; MDA, malondialdehyde; MIRI, myocardial ischemia–reperfusion injury; NOD, nucleotide-binding oligomerization domain; NYHA, New York Heart Association; OGD/R, oxygen–glucose deprivation/reoxygenation; PAMPs, pathogen-associated molecular patterns; PCI, percutaneous coronary intervention; PE, phosphatidylethanolamine; PUFAs, Polyunsaturated fatty acids; ROS, reactive oxygen species; STEAP3, Six-transmembrane epithelial antigen of prostate 3; STEMI, ST-segment elevation myocardial infarction; TAS, total antioxidant status; T2DM, type 2 diabetes mellitus; TFR1, transferrin receptor 1.
